# SGLT2 Inhibitors and GLP-1 Receptor Agonists in PAD: A State-of-the-Art Review

**DOI:** 10.3390/jcm14155549

**Published:** 2025-08-06

**Authors:** Alfredo Caturano, Damiano D’Ardes, Paola Giustina Simeone, Gianfranco Lessiani, Nicoletta Di Gregorio, Lorenzo Andreetto, Davide Grassi, Carla Serra, Francesca Santilli, Maria Teresa Guagnano, Fabio Piscaglia, Claudio Ferri, Francesco Cipollone, Andrea Boccatonda

**Affiliations:** 1 Department of Human Sciences and Promotion of the Quality of Life, San Raffaele Roma Open University, 00166 Rome, Italy; 2 Institute of “Clinica Medica”, Department of Medicine and Aging Science, “G. d’Annunzio” University of Chieti-Pescara, 66100 Chieti, Italypaola.simeone@unich.it (P.G.S.); guagnano@unich.it (M.T.G.);; 3 Angiology Unit, Internal Medicine Department, Città S. Angelo Hospital, Città S. Angelo, 65013 Pescara, Italy; 4 Department of Life, Health & Environmental Sciences and Internal Medicine, ASL Avezzano-Sulmona-L’Aquila, San Salvatore Hospital, University of L’Aquila, 67100 L’Aquila, Italy; 5 Internal Medicine, IRCCS Azienda Ospedaliero-Universitaria Policlinico di Sant’Orsola, 40138 Bologna, Italy; 6 Internal Medicine Unit, Val Vibrata Hospital-Sant’Omero (TE), Department of Life, Health and Environmental Sciences, University of L’Aquila, 67100 L’Aquila, Italy; 7 Diagnostic and Therapeutic Interventional Ultrasound Unit, IRCCS Azienda Ospedaliero-Universitaria di Bologna, 40138 Bologna, Italy; carla.serra@aosp.bo.it (C.S.);; 8 Department of Medical and Surgical Sciences, University of Bologna, 40126 Bologna, Italy; 9 Division of Internal Medicine, Hepatobiliary and Immunoallergic Diseases, IRCCS Azienda Ospedaliero-Universitaria di Bologna, 40138 Bologna, Italy

**Keywords:** sodium–glucose co-transporter-2 inhibitors (SGLT2is), GLP-1 receptor agonists (GLP-1 RAs), type 2 diabetes mellitus (T2DM), peripheral artery disease (PAD), cardiovascular outcomes, amputation risk

## Abstract

Sodium–glucose co-transporter-2 inhibitors (SGLT2is) and GLP-1 receptor agonists (GLP-1 RAs) are now established as cornerstone therapies for patients with type 2 diabetes mellitus (T2DM), given their cardiovascular and renal protective properties. However, their use in patients with peripheral artery disease (PAD) remains controversial due to concerns raised in early trials about potential increases in lower limb complications, particularly amputations. This narrative review examines current evidence on the association between SGLT2is and GLP-1 RAs in PAD-related outcomes, including limb events, amputation risk, and cardiovascular and renal endpoints. Drawing from randomized controlled trials, real-world cohort studies, and systematic reviews, we provide an integrated perspective on the safety and utility of SGLT2is and GLP-1 RAs in individuals with PAD, highlight patient selection considerations, and identify areas for future investigation.

## 1. Introduction

Peripheral artery disease (PAD) is a common comorbidity in individuals with type 2 diabetes mellitus (T2DM), conferring elevated risks of major adverse cardiovascular events (MACEs), limb ischemia, and amputation [1,2]. While glucose lowering is essential in managing T2DM, therapeutic strategies must also address macrovascular complications [3]. The introduction of sodium–glucose co-transporter-2 inhibitors (SGLT2is) and GLP-1 receptor agonists (GLP-1 RAs) has revolutionized diabetes care, offering consistent cardiovascular and renal benefits [3,4,5,6,7]. Yet, initial safety issues from the CANVAS trial raised concerns about an increased risk of amputation, prompting regulatory alerts and hesitation among clinicians to prescribe these agents to PAD patients [6,7,8]. As evidence has evolved, however, a more nuanced understanding has emerged—one that warrants careful re-evaluation and synthesis (Table 1).

This narrative review examines current evidence on the association between SGLT2is and GLP-1 RAs in PAD-related outcomes, including limb events, amputation risk, and cardiovascular and renal endpoints. Drawing from randomized controlled trials, real-world cohort studies, and systematic reviews, we provide an integrated perspective on the safety and utility of SGLT2is and GLP-1 RAs in individuals with PAD, highlight patient selection considerations, and identify areas for future investigation.

## 2. Literature Search Strategy

We conducted a targeted literature search to identify key studies examining the role of GLP-1 receptor agonists and SGLT2 inhibitors in patients with peripheral artery disease (PAD), including high-risk subgroups. PubMed, Embase, and the Cochrane Library were searched for English-language articles published from January 2010 to May 2025 using terms such as “peripheral artery disease”, “GLP-1 receptor agonists”, “SGLT2 inhibitors”, “diabetic foot ulcer”, and “major adverse limb events”.

We focused on clinical trials and observational studies reporting limb-specific outcomes or addressing high-risk PAD populations specifically in patients with diabetes. Review articles, case reports, and studies with incomplete data were excluded. The selected literature was synthesized narratively to highlight relevant clinical implications and knowledge gaps.

## 3. Antidiabetic Medications in PAD: Current Guidelines

Recent guidelines from the 2024 European Society of Cardiology (ESC) [9] and the 2024 American College of Cardiology/American Heart Association (ACC/AHA) guidelines [10] highlight the importance of using antidiabetic medications with proven cardiovascular benefits in managing patients with T2DM and PAD (Table 2). The ESC 2024 guidelines recommend the use of GLP-1 RAs and SGLT2is in patients with T2DM and PAD to reduce cardiovascular events, regardless of HbA1c levels or concurrent glucose-lowering therapy [9]. This recommendation is given Class I, Level of Evidence A [9]. Although the role of these medications in patients with prediabetes and established macrovascular disease remains unclear, glycemic control is still considered essential in T2DM with PAD [9]. Notably, some studies have suggested a possible increased risk of lower limb amputation with SGLT2is, especially canagliflozin, though the evidence is conflicting and warrants further investigation [8,9]. Similarly, the ACC/AHA 2024 guidelines also strongly advocate for the use of GLP-1 RAs and SGLT2is in PAD patients with T2DM to reduce the risk of MACEs, assigning this recommendation Class I, Level of Evidence A [10]. Furthermore, they highlight the importance of coordinated, multidisciplinary diabetes care (Class I, Level C—Expert Opinion) to optimize outcomes [10]. For limb-specific outcomes, they provide a Class IIb, Level B—Nonrandomized Evidence rating, suggesting that glycemic control may improve lower extremity outcomes, though the data are less conclusive than for cardiovascular benefit [10].

The 2023 intersocietal guidelines on PAD in individuals with diabetes, developed collaboratively by the IWGDF, ESVS, and SVS, provide important recommendations on the use of antidiabetic drugs in this high-risk population [11]. Among pharmacologic options, SGLT2is and GLP-1 RAs are recommended as part of the therapeutic strategy in individuals with T2DM and PAD, especially those with an estimated glomerular filtration rate (eGFR) above 30 mL/min/1.73 m^2^ [11]. These drugs have demonstrated cardiovascular benefits independent of their glucose-lowering effects and are thus endorsed in a “Best Practice Statement”. However, caution is advised when initiating SGLT2is in patients with active foot ulcers, gangrene, or infections, due to concerns about an increased risk of diabetic ketoacidosis and lower limb amputation, particularly in the case of canagliflozin [11]. Consequently, in drug-naïve individuals with foot ulcers, the initiation of SGLT2is is not recommended until the ulcer has healed [11]. In contrast, GLP-1 RAs are considered a safer option in the context of foot complications and may be preferable in these settings [11]. The guidelines also underline the importance of individualizing glycemic targets: while a target HbA1c of less than 8% is generally recommended, more intensive control (<7%) is not advised in patients with PAD and foot ulcers, particularly in the elderly or those at risk of hypoglycemia [11].

In summary, all guidelines support the integration of GLP-1 RAs and SGLT2is into the management strategy for patients with PAD and T2DM. These agents should be prioritized for their cardiovascular protective effects, with careful consideration of individual risk profiles, potential side effects, and the need for personalized care planning.

## 4. SGLT2is in PAD

### 4.1. Overview of SGLT2is

The SGLT2i is a class of oral antihyperglycemic agents that act independently of insulin by promoting urinary glucose excretion through the inhibition of SGLT2 in the renal proximal tubules [12,13]. Beyond glycemic control, they exert clinically significant cardiovascular and renal benefits, including reductions in blood pressure, weight, heart failure hospitalizations, and progression of kidney disease [14,15,16]. Clinical trials have consistently demonstrated that SGLT2 inhibitors provide significant cardiovascular protection, reducing rates of heart failure hospitalization and MACEs. Furthermore, their renal protective properties have positioned them as a cornerstone therapy not only for glucose control, but also for preserving kidney function in patients with and without diabetes [16].

### 4.2. Potential Mechanisms Underlying the Vascular Protective Effects of SGLT2is

While SGLT2is have demonstrated considerable cardiovascular and renal benefits in T2DM, their impact on vascular health, particularly concerning PAD and lower limb complications, is a subject of ongoing investigation [17]. The vascular effects of SGLT2is are complex, encompassing both protective and potentially harmful outcomes.

On the one hand, SGLT2is confer significant vascular protective effects through multiple mechanisms. By increasing nitric oxide (NO) bioavailability, reducing oxidative stress, and inhibiting endothelial cell apoptosis, SGLT2is enhance endothelial function, leading to improved vasodilation and vascular health [18,19]. This is supported by evidence of enhanced flow-mediated dilation (FMD) in patients treated with these agents [18]. Furthermore, SGLT2is have been shown to decrease arterial stiffness, as measured by pulse wave velocity (PWV), thereby improving arterial compliance and reducing the workload on the heart [20]. They also enhance the function of endothelial progenitor cells (EPCs) and facilitate the repair of a damaged endothelium, thereby supporting vascular regeneration and integrity [21]. Additionally, SGLT2is inhibit the proliferation and migration of vascular smooth muscle cells (VSMCs), key processes in the development of neointimal hyperplasia and vascular remodeling, which contributes to preventing vascular complications associated with PAD [21]. These agents also suppress inflammatory pathways by reducing pro-inflammatory cytokines such as interleukin-6 (IL-6) and tumor necrosis factor-alpha (TNF-α) [22], thus stabilizing atherosclerotic plaques and slowing atherosclerosis progression [22]. Moreover, by promoting natriuresis and reducing plasma volume, SGLT2is lower blood pressure and improve overall hemodynamic status, which is beneficial in reducing the risk of cardiovascular events in PAD patients [21]. It is important to acknowledge that many patients in the referenced studies were also receiving statins or other cardiovascular medications, which may independently exert vascular protective effects, particularly in reducing inflammation and atherosclerosis. While the specific contribution of SGLT2is can be difficult to isolate in such settings, emerging preclinical and mechanistic studies provide supportive evidence for direct vascular actions.

Recent preclinical studies, in fact, have deepened our understanding of SGLT2is’ vascular effects in PAD models [23]. These studies demonstrate enhanced nitric oxide synthase activity and reduced NADPH oxidase-derived reactive oxygen species (ROS), restoring redox balance in ischemic limbs [24,25]. Preservation of the endothelial glycocalyx supports vascular permeability and anti-inflammatory microcirculatory function [26,27]. Moreover, SGLT2is attenuate VSMC proliferation and neointimal hyperplasia and promote angiogenesis and tissue repair through improved EPC function and mitochondrial homeostasis, essential for regeneration following ischemic injury [28,29,30].

In summary, SGLT2 inhibitors confer vascular benefits in PAD through a multifaceted approach that includes improving endothelial function, exerting anti-inflammatory effects, reducing arterial stiffness, modulating VSMC activity, enhancing hemodynamic parameters, and promoting vascular repair mechanisms. These effects collectively contribute to the cardiovascular protective profile of SGLT2is in patients with PAD.

### 4.3. Potential Risks to Lower Limb Health with SGLT2is

However, despite these protective mechanisms, concerns have been raised regarding the potential adverse effects of SGLT2is on lower limb health [17]. The CANVAS trial highlighted a nearly twofold increase in the risk of lower limb amputations among patients treated with canagliflozin compared to a placebo [8]. This finding led the U.S. Food and Drug Administration (FDA) to issue a boxed warning for canagliflozin, which was later removed in 2020 after further evaluations [31]. Subsequent meta-analyses have suggested that this amputation risk may be specific to canagliflozin rather than a class-wide effect [32,33]. In EMPA-REG OUTCOME (empagliflozin) and DECLARE-TIMI 58 (dapagliflozin), no significant increase in amputation rates was reported, despite enrolling high-risk patients with cardiovascular disease or multiple risk factors [29,31]. Moreover, the CREDENCE trial, which also evaluated canagliflozin in patients with diabetic kidney disease, did not replicate the amputation signal seen in CANVAS, further adding complexity to the interpretation [34].

Several hypotheses have been proposed to explain this discrepancy. Canagliflozin may differ pharmacologically from other agents in the class, having greater off-target effects on SGLT1 inhibition, volume contraction, or bone mineral density [35,36], though direct evidence remains limited. Alternatively, differences may stem from study design, patient populations, or baseline risk factors. For instance, CANVAS included a high proportion of patients with prior amputations or severe PAD, which may have exaggerated risk in a susceptible subgroup [37].

Mechanistically, SGLT2is induce osmotic diuresis, leading to blood pressure reduction and hemoconcentration, potentially impairing peripheral perfusion—a concern in patients with pre-existing PAD [38,39]. Additionally, reduced tissue oxygenation and altered wound healing capacity may contribute to increased susceptibility to foot ulcers and infections, particularly in those with compromised microcirculation [39]. Ultimately, the net clinical effect may potentially depend on individual patient characteristics, such as baseline perfusion status, quality of foot care, and PAD severity [31].

Unfortunately, most cardiovascular outcome trials did not stratify or report detailed outcomes in PAD subgroups, making it difficult to draw firm conclusions. For example, while PAD prevalence was reported in some studies, such as ~20% in CANVAS and ~7–10% in DECLARE-TIMI 58 and EMPA-REG OUTCOME, event rates by PAD status were not uniformly analyzed [37,40,41]. As a result, the true interaction between SGLT2is and PAD-specific amputation risk remains uncertain. Further head-to-head trials and mechanistic studies are needed to clarify whether amputation risk is a true drug-specific phenomenon or reflects broader class-related effects under certain clinical conditions.

### 4.4. SGLT2is and PAD: Insights from Clinical Trials and Real-World Evidence

#### 4.4.1. Clinical Trials

The DECLARE-TIMI 58 trial provided critical insight into the impact of dapagliflozin on cardiovascular and limb outcomes among patients with and without PAD [40]. In the subgroup analysis, patients with PAD experienced significantly higher rates of MACEs and limb events compared to those without, but dapagliflozin did not considerably increase limb risk (HR for amputation 1.09; 95% CI 0.84–1.40) [40]. The CANVAS trial, by contrast, reported a nearly twofold increase in amputation risk with canagliflozin, raising early safety concerns [37]. However, subsequent trials, including EMPA-REG OUTCOME [41] and in a patient-level pooled analysis of the DAPA-HF and DELIVER trials, did not corroborate this signal [33]. Meta-analyses have sought to resolve these discrepancies. Geng et al. (2024) pooled 51 RCTs involving 97,589 patients and found a modest increase in PAD risk (OR 1.20; 95% CI 1.01–1.43; *p* = 0.04), but no significant increase in amputations (OR 1.18; 95% CI 0.78–1.79; *p* = 0.43) [42]. Subgroup analyses showed no differences by SGLT2i agent, treatment duration, or baseline risk profile, and found a modest increase in PAD risk (*p* = 0.04), but no significant increase in amputations (*p* = 0.43) [42]. Similarly, Nani et al. (2023) reported a neutral effect on PAD and osteomyelitis, but identified increased risks of lower limb ulcers (RR 1.39; 95% CI 1.01–1.91) and infections (RR 1.20; 95% CI 1.02–1.41) in SGLT2i users, based on a review of 42 RCTs [43]. Lin et al. (2021) highlighted a specific risk increase with canagliflozin, based on data from 40,925 users and 33,414 non-users [32]. The analysis showed elevated risks of amputation (OR 1.60; 95% CI 1.04–2.46) and PAD (OR 1.53; 95% CI 1.14–2.05), possibly linked to greater reductions in blood pressure and body weight. Particularly, lower baseline diastolic blood pressure (β = −0.528, 95% CI −0.852 to −0.205), a larger systolic blood pressure reduction (β = −0.207, 95% CI −0.390 to −0.023), and larger diastolic blood pressure reduction (β = −0.312, 95% CI −0.610 to −0.015) were significantly associated with the increased risks of amputation, PAD and diabetic foot, respectively, in patients with SGLT2i treatment. [32].

#### 4.4.2. Real-World Evidence

In a large U.S. Veterans cohort, Griffin et al. (2025) observed a higher rate of PAD-related surgical events, comparing 76,072 SGLT2i episodes to 75,833 DPP-4i episodes [44]. The event rates were 11.2 vs. 10.0 per 1000 people–years, with an adjusted HR of 1.18 (95% CI 1.08–1.29) [44]. In contrast, Taiwanese and Korean data suggested either neutral or protective effects [45]. For example, Lee et al. (2020) found that SGLT2is were associated with reduced risks of limb ischemia, amputation, and cardiovascular death compared to DPP-4is [46]. Similarly, Rodionov et al. (2021) noted that after the 2017 EMA safety warning, amputation risks between SGLT2is and GLP1-RAs equalized, suggesting that enhanced clinical monitoring may have mitigated early safety concerns [47].

A critical contribution came from Paul et al. (2021), who analyzed 3 million U.S. patients and found that PAD itself was the strongest predictor of lower limb amputation [48]. After propensity score adjustment, SGLT2i users had a lower relative risk of amputation compared to those on DPP-4is or other agents, and the conclusion was that the strongest predictor of amputation was PAD itself, rather than SGLT2i use [48]. The relative risk of lower limb amputation was lower in SGLT2i users compared to users of DPP-4is and other glucose-lowering agents after propensity score adjustments [48]. Patients with PAD who undergo revascularization represent a particularly high-risk group. Lee et al. (2023) evaluated outcomes in patients with prior PAD surgery and found that SGLT2is were not associated with increased risks of amputation or re-intervention, but did confer benefits in terms of reduced cardiac mortality and renal outcomes [49]. These findings support the continued use of SGLT2is even after limb interventions [49] (Table 3).

## 5. GLP1-RAs and PAD

GLP-1 RAs are a class of glucose-lowering agents that have gained recognition for their cardiovascular protective effects in T2DM. Beyond glycemic control, GLP-1 RAs exert a wide range of pleiotropic effects that may influence vascular health, including anti-inflammatory actions, endothelial protection, and the modulation of atherosclerotic pathways [50]. Given the high burden of PAD among patients with T2DM, understanding the impact of GLP-1 RAs on limb outcomes is of particular importance.

### 5.1. Potential Mechanisms Underlying the Vascular Protective Effects of GLP-1 RAs

GLP-1 RAs offer vascular protection through multiple interrelated mechanisms, extending far beyond their glucose-lowering effects [51]. One of the primary pathways involves endothelial protection. GLP-1 RAs enhance endothelial function by increasing NO bioavailability. This occurs through the activation of endothelial nitric oxide synthase, which promotes NO production, leading to improved vascular relaxation and reduced vascular stiffness [52]. Enhanced NO availability also protects against oxidative stress by neutralizing reactive oxygen species, thereby maintaining endothelial integrity [53]. In addition to their endothelial benefits, GLP-1 RAs exert potent anti-inflammatory effects. They inhibit the expression of pro-inflammatory cytokines such as IL-6, TNF-α, and C-reactive protein (CRP), which are directly implicated in the pathogenesis of atherosclerosis [54]. These anti-inflammatory properties contribute to plaque stabilization and prevent plaque rupture, a critical event in the progression of PAD. GLP-1 RAs also demonstrate favorable effects on VSMCs. By inhibiting VSMC proliferation and migration, they counteract neointimal hyperplasia, a process that underlies restenosis and vascular remodeling [51,55]. This effect is particularly relevant for patients with PAD, where abnormal VSMC behavior can exacerbate arterial narrowing [56]. Moreover, GLP-1 RAs enhance vascular repair mechanisms. They increase the mobilization and function of EPCs, which are essential for repairing a damaged endothelium [52]. This regenerative capacity further reinforces vascular integrity, reducing the risk of ischemic complications [57,58]. Another crucial aspect of GLP-1 RAs is their impact on hemodynamics. These agents reduce systolic and diastolic blood pressure, likely due to their natriuretic and diuretic effects mediated by GLP-1 receptors in the kidneys and by the activation of the afferent renal nerves [59,60]. Improved blood pressure control reduces arterial wall stress, enhances perfusion, and lowers the risk of MACEs [61]. Finally, GLP-1 RAs exhibit anti-atherosclerotic properties by modulating lipid metabolism [62]. They lower triglyceride levels and promote a favorable lipid profile, reducing the risk of atherosclerotic plaque formation [63].

Recent preclinical studies have expanded our understanding of the molecular mechanisms underpinning these vascular effects [64]. For example, liraglutide has demonstrated endothelial protective effects in ApoE−/− mice by downregulating plasminogen activator inhibitor type-1 (PAI-1) and vascular adhesion molecules, increasing eNOS expression, and reducing ICAM-1 levels [65]. In human vascular endothelial cells, liraglutide attenuates PAI-1, ICAM-1, and VCAM-1 expression induced by TNF-α or high glucose, possibly through the modulation of the transcription factor NUR77, which plays a key role in inflammation and endothelial stability [66,67]. Furthermore, liraglutide has been shown to reduce neointima formation by activating the AMPK–NO signaling pathway, protecting against endothelial-to-mesenchymal transition, an important process in diabetic vascular injury [68]. GLP-1 RAs also downregulate the NLRP3 inflammasome complex, a critical mediator of high glucose-induced endothelial dysfunction, by suppressing NLRP3, ASC, and caspase-1 expression and reducing oxidative stress via the inhibition of NADPH oxidase 4 (NOX4) in human umbilical vein endothelial cells [69]. Finally, GLP-1 RAs activate peroxisome proliferator-activated receptor gamma (PPARγ), which in turn inhibits intracellular kinases such as JNK and downregulates NF-κB signaling, thereby decreasing insulin resistance and limiting vascular inflammation. These effects have been demonstrated with exendin-4 treatment in endothelial cell models [70].

Collectively, these multifaceted actions establish GLP-1 RAs as potent agents for vascular protection, making them an attractive therapeutic option for patients with T2DM at risk of PAD and other cardiovascular complications.

### 5.2. GLP-1 RAs and PAD: Insights from Clinical Trials and Real-World Evidence

The cardiovascular and vascular benefits of GLP-1 RAs have been extensively studied in large, high-quality randomized controlled trials (RCTs). Among the most notable and recently published studies is the SOUL trial [71], a double-blind, placebo-controlled study involving 9650 participants aged 50 or older with type 2 diabetes (HbA_1_c 6.5–10.0%) and either atherosclerotic cardiovascular disease or chronic kidney disease. Over a median follow-up of just over four years, patients receiving oral semaglutide (up to 14 mg daily) experienced a 14% relative reduction in MACEs compared to the placebo (HR 0.86, 95% CI 0.77–0.96; *p* = 0.006). While the primary endpoint focused on MACEs, the trial underscored the cardiovascular safety of semaglutide without a significant impact on kidney outcomes. Building on this growing body of evidence demonstrating cardiovascular protection, there has been an increasing interest in exploring whether GLP-1 RAs can also confer benefits in reducing adverse limb outcomes. However, the data specific to limb-related endpoints remain limited and require cautious interpretation.

#### 5.2.1. Evidence from Randomized Controlled Trials (RCTs)

In the EXSCEL trial [72], which evaluated 2800 patients with PAD among a broader population with type 2 diabetes, exenatide treatment did not significantly reduce MACEs or lower-extremity amputation (LEA) rates compared to the placebo, regardless of PAD status. However, the trial highlighted the elevated risks of MACEs (aHR 1.13, 95% CI 1.00–1.27), all-cause mortality (aHR 1.38, 95% CI 1.20–1.60), and LEA (aHR 5.48, 95% CI 4.16–7.22) in patients with PAD compared to those without.

The STARDUST trial [73] explored the effects of liraglutide on peripheral perfusion in 55 patients with type 2 diabetes and PAD, using transcutaneous oxygen pressure (TcPo_2_) as a marker. Over six months, liraglutide significantly improved TcPo_2_ (mean difference: +11.2 mm Hg; *p* < 0.001), with 89% of liraglutide-treated patients achieving a ≥10% increase compared to 46% in the control group. Liraglutide also reduced markers of inflammation (C-reactive protein), improved renal function (albuminuria), and enhanced walking distance (+25.1 m; *p* < 0.001).

Another key trial, STRIDE [32], is a phase-3b, randomized, placebo-controlled trial assessing the impact of once-weekly semaglutide on functional outcomes in 792 patients with symptomatic PAD and type 2 diabetes. The trial demonstrated a significant improvement in maximum walking distance at week 52 in the semaglutide group compared to the placebo (estimated treatment ratio 1.13, 95% CI 1.06–1.21; *p* = 0.0004), highlighting the potential of semaglutide to enhance functional capacity in PAD patients.

#### 5.2.2. Insights from Real-World Evidence

Real-world studies further support the benefits of GLP-1 RAs in reducing adverse limb outcomes in patients with PAD. In a large retrospective cohort study of 948,342 patients with type 2 diabetes in Taiwan, Lin et al. identified 4460 GLP-1 RA users and 13,380 matched DPP-4i users [74]. GLP-1 RA therapy was associated with significantly lower rates of major adverse limb events (MALEs) (2.59 vs. 4.22 events/1000 person-years; SHR 0.63, 95% CI 0.41–0.96). The benefit was largely driven by a reduction in amputation rates (1.29 vs. 2.4 per 1000 people–years; SHR 0.55, 95% CI 0.30–0.99). GLP-1 RAs also reduced cardiovascular outcomes (HR 0.62, 95% CI 0.51–0.76), particularly in patients using statins and those without pre-existing cardiovascular disease.

In a multicenter retrospective cohort study using TriNetX data, Wu et al. analyzed 8046 propensity score-matched patients with PAD and type 2 diabetes [75]. Treatment with tirzepatide, a dual GLP-1 and GIP receptor agonist, significantly reduced the risk of MALEs (HR 0.44, 95% CI 0.33–0.59; *p* < 0.001) compared to controls. Tirzepatide was also associated with lower all-cause mortality, reduced stroke, and fewer MACEs, though the incidence of acute myocardial infarction (AMI) was similar between groups. These findings were consistent across most subgroups, except in patients with prior stroke.

#### 5.2.3. Current Limitations

Despite these encouraging findings, several limitations must be acknowledged. Most randomized controlled trials evaluating GLP-1 RAs were not specifically designed to assess limb-related outcomes, and lower-extremity events such as amputation or critical limb ischemia were often not predefined endpoints, or were reported post hoc. The sample sizes of PAD-specific subgroups were relatively small, reducing the statistical power to detect meaningful differences. Additionally, in real-world studies, residual confounding and differences in baseline limb risk (e.g., presence of foot ulcers, PAD severity, and revascularization history) may influence outcomes. Furthermore, the follow-up durations in many studies may be insufficient to capture the full trajectory of limb complications. Collectively, while current data are promising, more targeted and adequately powered studies with standardized limb-specific endpoints are needed to confirm the protective role of GLP-1 RAs in patients with PAD (Table 4).

## 6. Discussion

While both drug classes have demonstrated cardiovascular and renal benefits, their effects on limb-specific outcomes remain less well-defined (Table 5). The heterogeneity of the included studies, varying in design, patient populations, PAD definitions, and primary endpoints, poses a significant challenge to direct comparisons. To enhance interpretability, we have distinguished between randomized controlled trials and real-world evidence, noting differences in study quality and potential sources of bias. Although several observational studies suggest limb-protective effects, the absence of standardized outcome measures and confounder adjustment limits the strength of these conclusions. The concern about an increased risk of amputation with canagliflozin, first observed in the CANVAS trial, remains contentious [37]. Our review reflects this ongoing debate, noting that more recent analyses and trials of other SGLT2is (e.g., dapagliflozin, empagliflozin) have not consistently replicated this risk [41,47,48]. While RCTs offer high internal validity and have elucidated the potential cardiovascular and renal benefits of SGLT2is, their findings may not always be generalizable to broader patient populations. In contrast, real-world studies offer a complementary perspective by examining SGLT2is’ safety and efficacy across diverse clinical settings and patient profiles. However, it is crucial to recognize the inherent differences in methodological rigor and potential for bias between these study designs. RCTs reduce confounding and selection bias through randomization and blinding, but their strict protocols may limit external validity. Real-world evidence, often derived from observational cohorts or registry data, better reflects routine practice but is more susceptible to biases such as confounding by indication, missing data, and outcome misclassification. As such, findings from real-world studies should be interpreted cautiously and ideally assessed using structured tools such as ROBINS-I when comparing across evidence types.

This highlights the need for continued vigilance, as well as for more definitive, head-to-head studies assessing limb-specific safety outcomes across the drug class. GLP-1 RAs are generally viewed favorably in this context, with some data suggesting reductions in cardiovascular events and potential anti-inflammatory benefits. However, most available trials lack dedicated limb-specific endpoints, and much of the supporting evidence comes from post hoc analyses. Therefore, while promising, conclusions regarding their limb-related benefits should also be interpreted with caution.

## 7. Conclusions

SGLT2is and GLP-1 RAs have revolutionized the management of T2DM, providing significant cardiovascular and renal benefits beyond glycemic control. In patients with PAD, SGLT2is have shown promise in reducing cardiovascular events and slowing kidney disease progression, although the potential risk of limb complications, particularly with canagliflozin, necessitates careful patient selection and monitoring. Conversely, GLP-1 RAs have consistently demonstrated favorable effects on limb outcomes, including reduced rates of MALEs and amputations. Together, these agents represent a crucial advancement in the management of T2DM patients with concomitant PAD. However, optimizing their use requires a personalized approach, considering individual risk factors, comorbidities, and treatment goals. Continued research is essential to clarify the long-term impact of these therapies on limb outcomes, identify the patient subgroups most likely to benefit, and develop strategies for mitigating any associated risks.

## 8. Future Directions

While substantial evidence supports the cardiovascular benefits of SGLT2is and GLP-1 RAs in patients with type 2 diabetes, their specific impact on PAD remains insufficiently characterized (Table 5). Future research should prioritize large-scale RCTs specifically targeting PAD populations, with well-defined endpoints such as MALEs, LEA, and functional outcomes, including walking capacity and quality of life. Comparative studies are also needed to clarify the differential effects of SGLT2is and GLP-1 RAs on PAD progression. A critical gap in current evidence is the absence of head-to-head trials directly comparing these drug classes in patients with PAD, especially for outcomes like MALEs and wound healing. Mechanistic studies exploring how these agents modulate inflammation, endothelial function, and microvascular health would further enhance the understanding of their therapeutic potential [76].

Special attention should be given to high-risk subgroups, including patients with active diabetic foot ulcers or those who have recently undergone revascularization. For these populations, prospective cohort studies and pragmatic trials may offer valuable insights into safety, wound healing, and limb salvage. Additionally, registry-based studies could help to capture real-world effectiveness and long-term outcomes across diverse care settings (Table 6). Such targeted and comparative data will be essential to inform personalized treatment strategies and optimize vascular outcomes in patients with PAD and type 2 diabetes.

## Figures and Tables

**Table 1:** Comparison of antidiabetic drug classes in patients with peripheral artery disease.

Drug Class	Mechanism	CV Effect	Hypoglycemia Risk	Weight Effect	Comments in PAD
Metformin	Insulin sensitizer	Neutral	Low	Weight neutral or modest loss	Risk of lactic acidosis in advanced PAD
Sulfonylureas	Insulin secretagogue	Neutral/possibly adverse	Moderate	Weight gain	Risk of hypoglycemia; limited CV benefit
DPP-4 inhibitors	Incretin enhancer	Neutral	Low	Weight neutral	Safe but limited benefit in PAD
SGLT2 inhibitors	Glycosuria inducer	Benefit (CV and renal)	Low to moderate	Weight loss	Caution in active foot ulcers; canagliflozin amputation signal
GLP-1 receptor agonists	Incretin mimetic	Benefit (CV)	Low	Weight loss	Preferred in PAD with CV disease
Insulin	Direct glucose lowering	Neutral/possibly adverse	High	Weight gain	Risk of hypoglycemia; avoid overtreatment
Thiazolidinediones	Insulin sensitizer	Neutral/possibly adverse	High	Weight gain	Contraindicated in heart failure

CV: cardiovascular; PAD: peripheral artery disease; DPP-4: Dipeptidyl Peptidase-4; and SGLT2: sodium–glucose co-transporter-2.

**Table 2:** Guideline recommendations for the use of GLP-1 RAs and SGLT2is in patients with T2DM and PAD.

Guideline	Recommended Medications	Purpose	Class/Level of Evidence	Notes
ESC 2024 [9]	GLP-1 RAs, SGLT2is	Reduce CV events in T2DM and PAD	Class I, Level A	Recommended regardless of HbA1c or concurrent therapy
	—	Improve glycemic control	—	Essential in T2DM with PAD
	—	Potential risk: lower limb amputation	—	Mainly linked to canagliflozin; evidence conflicting
ACC/AHA 2024 [10]	GLP-1 RAs, SGLT2is	Reduce MACEs in T2DM and PAD	Class I, Level A	Strong recommendation
	Multidisciplinary diabetes care	Optimize overall outcomes	Class I, Level C (Expert Opinion)	Coordinated care encouraged
	Glycemic control	Improve limb outcomes	Class IIb, Level B (Nonrandomized Evidence)	Evidence less robust for limb-specific outcomes
IWGDF/ESVS/SVS 2023 [11]	SGLT2is, GLP-1 RAs	Reduce CV risk, improve PAD outcomes	Best Practice Statement	Use GLP-1 RAs preferentially in active foot ulcers
	SGLT2is (delayed initiation)	Avoid adverse outcomes in active DFU	Not recommended until ulcer healing	Risk of ketoacidosis/infection with SGLT2is in DFU
	Glycemic target (HbA1c < 8%)	Avoid hypoglycemia in high-risk patients	Best Practice Statement	Lower targets not recommended in elderly or those with foot ulcers

ACC/AHA: American College of Cardiology/American Heart Association; CV: cardiovascular; DFU: diabetic foot ulcer; ESC: European Society of Cardiology; ESVS: European Society for Vascular Surgery; GLP-1 RA: glucagon-like peptide-1 receptor agonist; IWGDF: International Working Group on the Diabetic Foot; MACEs: major adverse cardiovascular events; PAD: peripheral artery disease; SGLT2i: sodium–glucose co-transporter-2 inhibitor; SVS: Society for Vascular Surgery; and T2DM: type 2 diabetes mellitus.

**Table 3:** Summary of evidence on SGLT2 inhibitors and PAD/amputation risk.

Study/Source	Design/Population	Findings on PAD/Amputation Risk	Key Notes
DECLARE-TIMI 58 (2019) [40]	RCT; dapagliflozin (*n* = 17,160 patients with T2DM who either had established atherosclerotic cardiovascular disease or were at risk of it; about 10% with PAD)	No significant increase in amputation (HR 1.09; 95% CI 0.84–1.40)	PAD patients had higher baseline MACE/limb risk; dapagliflozin neutral
CANVAS Program (2018) [37]	RCT; canagliflozin (*n* = 10,142 patients with T2DM)	Nearly 2× increased amputation risk	Prompted early regulatory concern
EMPA-REG OUTCOME (2015) [41]	RCTs; empagliflozin (*n* = 7020 patients with T2DM and established ASCVD	No increased amputation risk	Supported safety reassurance
DAPA-HF + DELIVER (2022) [33]	Patient-level meta-analysis (*n* ≈ 11,000; 809 with PAD)	No increased amputation risk; PAD subgroup: dapagliflozin 3.7% vs. placebo 4.2%	No interaction between treatment and amputation risk in PAD
Geng et al. (2024) [42]	Meta-analysis of 51 RCTs (*n* = 97,589)	↑ PAD risk (OR 1.20; *p* = 0.04); no ↑ in amputation (OR 1.18; *p* = 0.43)	No differences by agent, duration, or baseline risk
Nani et al. (2023) [43]	Meta-analysis of 42 RCTs (n = 29,491 and 23,052 patients, respectively, assigned to SGLT2-i and comparator groups)	Neutral PAD risk; ↑ limb ulcers (RR 1.39), ↑ infections (RR 1.20)	Elevated soft tissue complication risks
Lin et al. (2021) [32]	Meta-analysis; 40,925 SGLT2i users vs. 33,414 non-users	↑ Amputation (OR 1.60), ↑ PAD (OR 1.53); esp. with canagliflozin	Risks linked to BP/weight reductions; strongest with lower DBP
Griffin et al. (2025) [44]	Real-world; U.S. Veterans (n ≈ 76,000/group)	↑ PAD-related surgeries (HR 1.18; 95% CI 1.08–1.29) vs. DPP-4is	Large sample; adjusted HR
Lee et al. (2020) [46]	Real-world; Taiwan (n = 11,431 and 93,972 consecutive T2DM patients with PAD taking SGLT2is and DPP4is, respectively)	↓ Limb ischemia, amputation, CV death vs. DPP-4is	Suggests net benefit in Asian cohort
Rodionov et al. (2021) [47]	Real-world; Europe, post-EMA safety alert (n = 44,284 (13.6% PAD) and 56,878 (16.3% PAD) T2DM patients initiated with SGLT2is or GLP1-RAs, respectively)	Amputation risk comparable between SGLT2is and GLP-1 RAs	Risk mitigation via clinical monitoring
Paul et al. (2021) [48]	U.S. observational; ~3 million patients	PAD = main amputation risk; SGLT2is had ↓ risk vs. DPP-4is/others	PAD, not SGLT2i, was primary risk driver
Lee et al. (2023) [49]	Real-world (n = 2455 and 8695 patients with T2DM who had undergone PAD revascularization and received first prescriptions for SGLT2is and DPP4i, respectively	No ↑ in amputation/re-intervention; ↓ CV and renal events	SGLT2is safe in high-risk surgical PAD group

SGLT2i: sodium–glucose co-transporter 2 inhibitor; PAD: peripheral artery disease; RCT: randomized controlled trial; HR: Hazard Ratio; OR: Odds Ratio; RR: Risk Ratio; CI: Confidence Interval; MACEs: major adverse cardiovascular events; CV: cardiovascular; DBP: diastolic blood pressure; BP: blood pressure; GLP-1 RA: glucagon-like peptide-1 receptor agonist; DPP-4i: Dipeptidyl Peptidase-4 Inhibitor; LLA: lower limb amputation; and EMA: European Medicines Agency.

**Table 4:** Summary of evidence on GLP-1 RA and PAD/amputation risk.

Study/Source	Design/Population	Findings on PAD/Amputation Risk	Key Notes
SOUL trial [71]	RCT; oral semaglutide; 9650 adults ≥50 yrs with T2D + ASCVD/CKD	↓ MACE by 14% (HR 0.86; 95% CI 0.77–0.96); no renal outcome effect	Focused on CV safety; limb outcomes not primary endpoint
EXSCEL trial [72]	RCT; exenatide; included ~2800 PAD patients	No ↓ in MACE or LEA; PAD patients had ↑ MACE, mortality, and LEA risk	aHR for LEA in PAD vs. non-PAD: 5.48 (95% CI 4.16–7.22)
STARDUST trial [73]	RCT; liraglutide; 55 T2D patients with PAD	↑ TcPo_2_ (+11.2 mm Hg); 89% had ≥10% increase vs. 46% in control	Also improved CRP, albuminuria, and walking distance (+25.1 m)
STRIDE trial [32]	RCT; semaglutide; 792 PAD patients with T2D	↑ Maximum walking distance (ETR 1.13; 95% CI 1.06–1.21; *p* = 0.0004)	Showed functional improvement in PAD patients
Lin et al. (2021) [74]	Real-world; 17,840 patients (GLP-1 RA vs. DPP-4i), Taiwan	↓ MALEs (SHR 0.63); ↓ amputations (SHR 0.55); ↓ CV outcomes (HR 0.62)	Benefit most evident in statin users and those w/o prior CVD
Wu et al. (2025) [75]	Real-world; 8046 matched PAD patients with T2D	↓ MALEs (HR 0.44); ↓ stroke, mortality, and MACE; AMI risk unchanged	Tirzepatide = GLP-1/GIP RA; effects consistent across most subgroups

RCT: randomized controlled trial; T2D: type 2 diabetes; ASCVD: atherosclerotic cardiovascular disease; CKD: chronic kidney disease; MACEs: major adverse cardiovascular events; HR: Hazard Ratio; CI: Confidence Interval; CV: cardiovascular; LEA: lower-extremity amputation; PAD: peripheral artery disease; aHR: Adjusted Hazard Ratio; TcPo_2_: transcutaneous oxygen pressure; CRP: C-reactive protein; ETR: estimated treatment ratio; MALEs: major adverse limb events; SHR: Subdistribution Hazard Ratio; CVD: cardiovascular disease; GLP-1 RA: glucagon-like peptide-1 receptor agonist; DPP-4i: Dipeptidyl Peptidase-4 Inhibitor; GIP: Glucose-Dependent Insulinotropic Polypeptide; and AMI: acute myocardial infarction.

**Table 5:** Advantages and limitations of GLP-1 RAs vs. SGLT2is in patients with PAD.

Therapy	Advantages	Limitations	PAD with DFU	PAD without DFU
GLP-1 RAs	- CV risk reduction - Weight loss benefit - Potential anti-inflammatory effects	- Gastrointestinal side effects (nausea, vomiting) - Injectable formulation (except oral semaglutide) - Limited data on limb outcomes	- Generally safe; no increase in amputation risk - Useful in obese patients with PAD and DFU	- May reduce MACE risk - No clear benefit on limb events in trials
SGLT2is	- CV and renal protection - Oral administration - Reduction in heart failure hospitalizations	- Risk of volume depletion - Increased risk of genital infections - Potential amputation risk (controversial, mainly with canagliflozin)	- Use with caution in patients at high risk of foot ulcers or lower-limb complications - Avoid in patients with active infections	- Beneficial for CV and renal outcomes - Monitor hydration status and foot care

PAD = peripheral artery disease; DFU = diabetic foot ulcer; MACEs = major adverse cardiovascular events; CV = cardiovascular; GLP-1 RAs = glucagon-like peptide-1 receptor agonists; SGLT2is = sodium–glucose co-transporter 2 inhibitors; and GI = gastrointestinal.

**Table 6:** Research questions and suggested study designs in PAD Subgroups.

Objective	Design	Key Features
Compare Limb Outcomes: GLP-1 RAs VS. SGLT2is	Randomized Controlled Trial (RCT)	Multicenter, stratified by PAD severity; primary endpoint: MALEs
Assess Safety in Active Foot Ulcers	Prospective Cohort Study	Track wound healing, infection rates, and amputation in patients initiating therapy
Post-Revascularization Benefit	RCT or Pragmatic Trial	Randomize patients after revascularization; endpoints: reocclusion, amputation, MALEs
Evaluate Combined Therapy	Factorial RCT	2 × 2 design: GLP-1 RA, SGLT2i, both, or neither
Real-World Outcomes in High-Risk Groups	Registry-Based Study	Use PAD/diabetes registries to evaluate long-term limb and CV outcomes by treatment class

PAD = peripheral artery disease; MALEs = major adverse limb events; GLP-1 RAs = glucagon-like peptide-1 receptor agonists; SGLT2is = sodium–glucose co-transporter 2 inhibitors; and CV = cardiovascular.

## Data Availability

No dataset was generated for the publication of this article.
